# A meta‐analysis of toxicities related to hydroxycarbamide dosing strategies

**DOI:** 10.1002/jha2.7

**Published:** 2020-04-26

**Authors:** Joacy G. Mathias, Vikki G. Nolan, Meghan Meadows‐Taylor, L. Ashley Robinson, Kristen E. Howell, James G. Gurney, Jane S. Hankins, Winfred C. Wang, Jeremie H. Estepp, Matthew P. Smeltzer

**Affiliations:** ^1^ Division of Epidemiology, Biostatistics, and Environmental Health School of Public Health The University of Memphis Memphis Tennessee; ^2^ Department of Hematology St. Jude Children's Research Hospital Memphis Tennessee; ^3^ Department of Pathology St. Jude Children's Research Hospital Memphis Tennessee

**Keywords:** dosing, hydroxycarbamide, meta‐analysis, sickle cell disease

## Abstract

Due to fear of short‐term toxicities, there is nonconsensus of hydroxycarbamide dosing strategy (escalated vs fixed‐dosing methods), which contributes to its suboptimal use. We performed a meta‐analysis to summarize the incidence rates of toxicities associated with both dosing methods. Summarized incidence rates could not be statistically compared between dosing methods due to sparse data. Summarized neutropenia and thrombocytopenia incidence rates were slightly higher when using escalated dosing than with fixed. Summarized reticulocytopenia was comparable. Summarized hepatic and renal toxicities’ incidence rates were slightly higher when using fixed doses than with escalated. We recommend diligent and transparent reporting of toxicities.

## 1 INTRODUCTION

Hydroxycarbamide (also known as hydroxyurea [HC]) is an approved treatment for adults and children (>2 years of age) with sickle cell anemia (SCA; HbSS and HbSβ^0^ thalassemia) [7]. Based on the randomized, placebo‐controlled, multicenter, BABY HUG trial (PMID: 21571150), HC is also commonly prescribed off‐label by pediatric hematologists for infants (9‐18 months old) (PMID: 20223921). Despite HC being front‐line therapy for children and adults with SCA, there is a paucity of high‐quality evidence regarding toxicity rates observed when employing two accepted dosing strategies: minimum effective dosing and maximum tolerated dosing [1, 3]. Toxicity data are of critical importance as novel anti‐sickling agents such as Oxbryta (voxelotor) and Adakveo (crizanlizumab) have been developed in the setting of widespread concomitant use of HC. We performed a meta‐analysis comparing myelosuppressive, renal, and hepatic toxicities among minimum effective (fixed) dosing and maximum tolerated dosing protocols reported in published studies between 2010 and 2015. We censored selection of reports from after 2010 in an effort to ensure data captured represented modern sickle cell cohorts and reflected current standards of medical care and from prior to 2015 to exclude studies performed in sub‐Saharan Africa where toxicity profiles of hydroxycarbamide may be exaggerated.

## 2 METHODS

Studies for potential inclusion were identified by searching PubMed and Elsevier's Scopus electronic databases. The search criteria used were the same as those of the National Heart, Lung, and Blood Institute (NHLBI) in a comprehensive evaluation of HC as a treatment for SCD (http://www.ncbi.nlm.nih.gov/books/NBK38496/). Full‐text articles published in English, between 2010 and 2015 with a study design of either a randomized or nonrandomized clinical trial, or a prospective or retrospective cohort, and reporting at least myelosuppressive, renal, or hepatic toxicity information were included. In the event of multiple published reports from the same study, we included the report with more information.

Myelosuppressive toxicities were defined as (a) Neutropenia: absolute neutrophil count of <1.0 × 10^9^/L, (b) Anemia: hemoglobin (Hb) <6.0 g/dL with concomitant absolute reticulocyte count <80 × 10^9^/L, (c) Reticulocytopenia: absolute reticulocyte count <80 × 10^9^/L associated with an Hb level <9.0 g/dL, (d) Thrombocytopenia: platelet count <80 × 10^9^/L, (e) Renal toxicity: doubling of the baseline serum creatinine level or a value of >1.0 mg/dL, and (f) Hepatic toxicity: increase in alanine aminotransferase to ≥150 IU/L. Due to variation in the definitions of toxicities across studies, we also included author‐defined toxicities.

Two independent reviewers and experienced hematologists (JHE and WCW) selected eligible studies for inclusion. In the event of disagreement between JHE and WCW, JSH provided an independent assessment of inclusion criteria and served as a tie‐breaker. Following manuscript selection, relevant data were abstracted including the HC dosage method, sample size, total person‐years of follow‐up, number of study participants with HC exposure, ages and genotypes of participants, and frequency of toxicities.

Incidence rates (*Y*_i_/*T*_i_) of each toxicity, along with 95% confidence intervals (CIs), were calculated by dividing the frequency of reported toxicities (*Y*_i_) with the exposure patient years (PY) of follow‐up (*T*_i_) for each study. Using a random intercept Poisson regression model (Proc NLMIXED), incidence rates were averaged to calculate the summarized incidence rate for studies that employed fixed dosing and those that employed escalated dosing separately [11]. In post hoc sensitivity analyses, data from three studies deemed as outliers, that is, those with remarkably high frequency of toxicities were excluded and summarized incidence rates recalculated. All analyses were performed using the Statistical Analysis System (SAS) statistical software package version 9.4 (SAS Institute Inc., Cary, NC, USA).

## 3 RESULTS

**FIGURE 1:**
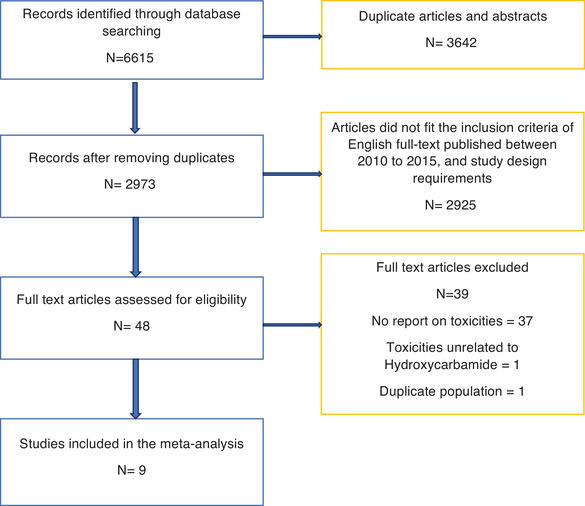
Study Inclusion Flow Chart

Nine studies, described in Supplementary table 1, were included in the quantitative analysis. Figure 1 shows the Preferred Reporting Items for Systematic Reviews and Meta‐Analyses flow chart for study inclusion. Two studies were multi‐center. Included studies were conducted in the United States, Brazil, and India. One study was a randomized clinical trial. The majority of the studies were funded by the NHLBI. Five studies escalated HC to maximum tolerated dosing and provided 1849 PY of HC exposure with 161 reported toxicities, and four studies used a fixed dosing strategy and provided 848 PY of HC exposure with 149 reported toxicities.

The summarized incidence rate for neutropenia was 3.69 (95% CI, 1.56‐8.72) per 100 PY for studies utilizing escalation to maximum tolerated dose and 1.76 (95% CI, 0.14‐22.7) per 100 PY in studies utilizing a fixed dose (Table 1). The summarized incidence rate for neutropenia without data from the very young population in BABY HUG was 0.79 (95% CI, 0.23‐2.73) per 100 PY for fixed dosing (Supplementary table 2).

**TABLE 1:** Summary of Incidence Rates of Toxicities by Dosing Strategy

	Incidence rate/100 PY (95% CI)					
	Neutropenia	Anemia	Reticulocytopenia	Thrombocytopenia	Renal toxicity	Hepatic toxicity
MTD dosing strategy						
Hankins et al., 2015 [6]	4.44 (0‐13.16)	‐	4.44 (0‐13.16)	0	‐	‐
Hankins et al., 2014 [5]	8.33 (3.17‐13.50)	0.83 (0‐2.47)	0.83 (0‐2.47)	1.67 (0‐3.98)	0	0
Lobo et al., 2013 [9]	0.83 (0.36‐1.29)	‐	0.07 (0‐0.20)	0	0	0
Yates et al., 2013 [14]	4.44 (0.08‐8.80)	‐	‐	5.56 (0.69‐10.43)	0	0
Alvarez et al., 2015 [2]	7.88 (3.60‐12.16)	23.64 (16.22‐31.05)	18.18 (11.68‐24.69)	5.45 (1.89‐9.02)	4.24 (1.10‐7.39)	15.15 (9.21‐21.09)
Summary	3.69 (1.56‐8.72)	4.81 (0.39‐58.10)	1.23 (0.13‐11.71)	0.72 (0.07‐7.80)	0.02 (0‐38.80)	0 (0‐201.43)
Fixed dosing strategy						
Dehury et al., 2015 [4]	0.29 (0‐0.86)	‐	‐	1.17 (0.02‐2.31)	‐	‐
Patel et al., 2014 [10]	‐	‐	0	0	0	0
Jain et al., 2013 [8]	1.74 (0.21‐3.26)	0.53 (0‐1.57)	0.53 (0‐1.57)	1.39 (0.03‐2.75)	1.04 (0‐2.22)	2.78 (0.85‐4.70)
Wang et al., 2011 [12]	56.61 (45.89‐67.34)	‐	‐	6.35 (2.76‐9.94)	0	1.59 (0‐3.38)
Summary	1.76 (0.14‐22.74)	‐	0.21 (0.01‐4.93)	1.96 (0.75‐5.05)	0.61 (0.20‐1.86)	2.16 (1.19‐3.91)

Summarized incidence rates for anemia could not be calculated due to lack of information among studies that used fixed dosing. The summarized incidence rate for reticulocytopenia with escalated dosing was 1.23 (95% CI, 0.13‐11.71) per 100 PY and with fixed dosing 0.21 (95% CI, 0.01‐4.93) per 100 PY (Table 1). The summarized incidence rate for reticulocytopenia, excluding data from Alvarez et al, when using escalated dosing was 0.36 (95% CI, 0.05‐2.98) per 100 PY (Table S2).

The summarized incidence rate for thrombocytopenia when using escalated dosing was 0.72 (95% CI, 0.07‐7.79) per 100 PY, and for fixed dosing 1.96 (95% CI, 0.75‐5.05) per 100 PY (Table 1).

All studies except Alvarez et al and Jain et al reported no renal toxicities. The summarized incidence rate for renal toxicities when using escalated dosing was 0.02 (95% CI, 0‐38.80) per 100 PY, and for fixed dosing the summarized incidence rate was 0.61 (95% CI, 0.20‐1.86) per 100 PY (Table 1).

The summarized incidence rate for hepatic toxicities with escalated dosing was 0 (95% CI, 0‐201.43) per 100 PY, and for fixed dosing 2.16 (95% CI, 1.19‐3.91) per 100 PY (Table 1). The summarized incidence rate for hepatic toxicities when using escalated dosing strategy without Alvarez et al data was 0 (95% CI, 0‐0) per 100 PY (Table S2).

## 4 DISCUSSION

In this meta‐analysis, incidence rates of neutropenia and thrombocytopenia in the post hoc analyses appear higher when escalating HC to a maximum tolerated dose compared with utilizing a lower fixed dose; however, reticulocytopenia was comparable between the two dosing strategies. The incidence rates of hepatic and renal toxicities were slightly higher when using fixed dosing when compared with escalated doses. However, the results of our analysis were limited by the small number of studies reporting toxicity‐related information, imprecisely summarized incidence rates, and high heterogeneity between the studies, thus affecting our ability to draw conclusions about differences in toxicities related to the two HC dosing strategies.

For our meta‐analysis, only nine studies had information related to the toxicities. No information on toxicities was reported in 37 potentially eligible articles. Even among the studies with information, details on many specific toxicities were missing. Due to the myelosuppressive nature of HC, standard baseline measurements before its administration should have included a full blood count, liver function tests, and urea and creatinine levels [15]. Therefore, information related to the toxicities may be available but not reported due to a lack of relevance for a particular publication. Some studies indicated the incidence of HC‐related toxicities, but not the number of subjects with adverse events. The number of events and number of study participants with toxicities, irrespective of the relevance to the publication, may provide valuable information for future systematic reviews.

Several of the summarized toxicity incidence rates had wide 95% CIs. For instance, the summarized neutropenia incidence rate for studies that used a fixed dosing strategy was 1.76 per 100 PY (95% CI, 0.14‐22.74). The highest frequency of neutropenia among fixed dosing studies came from the BABY HUG trial [12], which increased the 95% CI of the summarized neutropenia incidence. HC dosing of 20 mg/kg/day in children who were 9‐18 months old at treatment initiation probably was associated with increased neutropenia because of the high frequency of viral infections in this very young age group [13]. Upon exclusion of these data, the 95% CI was relatively precise.

There were several sources of heterogeneity in our meta‐analysis, such as age and genotype. We tried to address this issue by using random‐effect modeling; however, heterogeneity persisted. We could not explore the sources of heterogeneity due to the small number of studies in escalated and fixed dosing groups and lack of information on toxicities by genotype and age group.

The lack of consensus regarding dosing strategy due to concern for short‐term toxicities may lead to suboptimal use of HC. To address this critical gap in the literature, we attempted a meta‐analysis comparing the toxicities associated with escalated and fixed dosing methods; however, we were unable to make conclusions on toxicities except neutropenia, mainly due to the lack of complete and systematic information on toxicities in many reports. Moving forward, we recommend diligent and standardized reporting of toxicities to help better understand the clinical effects of different dosing strategies.

## AUTHOR CONTRIBUTIONS

JGM conducted the statistical analysis, interpreted the data, and drafted the manuscript. VGN supervised the statistical analyses and aided in its interpretation, and critical revision of the manuscript. MMT, LAR, and KEH helped with the data collection, statistical analysis, and its interpretation. JGG and JSH supervised the statistical analyses and aided in its interpretation and revision of the manuscript. WCW and JHE applied for the grant and contributed to conception and design, data collection, interpretation, and revision of the manuscript. MPS contributed to the conception and design of the study, statistical analysis, interpretation, and critical revision of the manuscript.

## CONFLICT OF INTERESTS

JHE receives research support from Pfizer, Eli Lilly and Co, Global Blood Therapeutics, and Forma Therapeutics and serves as a consultant for Daiichi Sankyo and Global Blood Therapeutics. JSH receives research support through the grant U01 HL‐133996, and from Novartis and Global Blood Therapeutics, and received consultant fees from MJH Life Sciences. MPS is a research consultant for the Association of Community Cancer Centers.

## Supporting information

Supporting Information
