# Supplementary material for: A meta‐analysis of toxicities related to hydroxycarbamide dosing strategies
Source: EJHaem. 2020 Apr 26;1(1):235–8. doi: 10.1002/jha2.7 (PMC9176148; doi:10.1002/jha2.7)
Supplement: Supplementary file 1 — Supporting Information [file JHA2-1-235-s001.docx]

| **Author, Year** | **Genotype** | **Sample Size** | **Follow Up, y** | **Age Range, y** | **HC Dosage type** | **HC**  **ExposurePY** | **Frequency of toxicities** | | | | | |
| --- | --- | --- | --- | --- | --- | --- | --- | --- | --- | --- | --- | --- |
|  |  |  |  |  |  |  | **Neutro-penia** | **Anaemia** | **Reticulo-cytopenia** | **Thrombo-cytopenia** | **Renal** | **Hepatic** |
| Hankins, 2015 (Hankins*, et al* 2015) | HbSS, HbSβ0, HbSOArab, HbSD | 22 | 2.5 | 2 to 11 | Escalated | 22.5 | 1 | Not  given | 1 | 0 | Not  given | Not  given |
| Hankins, 2014 (Hankins*, et al* 2014) | HbSS | 8 | 15 | 15.5 to 16 | Escalated | 120.0 | 10 | 1 | 1 | 2 | 0 | 0 |
| Lobo, 2013 (Lobo*, et al* 2013) | HbSS, HbS β0, HbSβ+, HbSC, HbSD | 1760 | 3 to 17 | 3 to 18 | Escalated | 1451.6 | 12 | Not  given | 1 | 0 | 0 | 0 |
| Yates, 2013 (Yates*, et al* 2013) | HbSC | 15 | 3.1 to 13.2 | 3.8 to 14.9 | Escalated | 90.0 | 4 | Not  given | Not  given | 5 | 0 | 0 |
| Alvarez, 2013 (Alvarez*, et al* 2013) | HbSS, HbSβ0, HbSOArab | 133 | 2.5 | 5.2 to 19 | Escalated | 165.0 | 13 | 39 | 30 | 9 | 7 | 25 |
| Dehury, 2015 (Dehury*, et al* 2015) | HbSβ+ | 104 | ~2 | 19 to 32 | Fixed | 343.0 | 1 | Not  given | Not  given | 4 | Not  given | Not  given |
| Patel, 2014 (Patel*, et al* 2014) | HbSDPunjab | 20 | 2 | 1 to 45 | Fixed | 28.0 | 0 | Not  given | Not  given | 0 | 0 | 0 |
| Jain, 2013 (Jain*, et al* 2013) | HbSOArab | 144 | 2 | 3.5 to 17.9 | Fixed | 288.0 | 5 | Not  given | 0 | 4 | 3 | 8 |
| Wang, 2011 (Wang*, et al* 2011) | HbSS, HbSβ0 | 193 | 2 | 0.8 to 1.5 | Fixed | 189.0 | 107 | 1 | 1 | 12 | 0 | 3 |

**Supplementary Table 1. Included Study Characteristics**

**Supplementary Table 2. Summaries of Laboratory Toxicities Using Selective Data**

| **Toxicities** | **Escalated IR/100 PY (95%CI)** | **Escalated - across study variance** | **Fixed IR/100 PY (95%CI)** | **Fixed-across study variance** |
| --- | --- | --- | --- | --- |
| Neutropenia with Wang *et al*., 2011 data | 3.69 (1.56, 8.72) | 0.73 | 1.76 (0.14, 22.74) | 5.25 |
| Neutropenia without Wang *et al*., 2011data | 3.69 (1.56, 8.72) | 0.73 | 0.79 (0.23, 2.73) | 0.28 |
| Thrombocytopenia with Wang *et al*., 2011 data | 0.72 (0.07, 7.80) | 0.00 | 1.96 (0.75, 5.05) | 0.00 |
| Thrombocytopenia without Lobo *et al*., 2013 and Wang *et al*., 2011 data | 4.01 (2.27, 7.10) | 0.04 | 1.23 (0.61, 2.29) | 0.00 |
| Reticulocytopenia with Alvarez *et al*., 2013 data | 1.23 (0.13, 11.71) | 4.42 | 0.21 (0.01, 4.93) | 0.00 |
| Reticulocytopenia without Alvarez *et al*., 2013 data | 0.36 (0.05, 2.98) | 1.85 | 0.21 (0.01, 4.93) | 0.00 |
| Hepatic toxicities with Alvarez *et al*., 2013 data | 0 (0, 201.43) | 65.64 | 2.16 (1.19, 3.91) | 0.00 |
| Hepatic toxicities without Alvarez *et al*., 2013 data | 0 (0) | 0 | 2.16 (1.19, 3.91) | 0.00 |
